# Mental Derangement, Its Symptoms and Treatment

**Published:** 1855-01

**Authors:** Roger G. Perkins

**Affiliations:** Senior Assistant Physician, New York Lunatic Asylum, Blackwell’s Island


### DEPARTMENT OF SELECTIONS.

### MENTAL DERANGEMENT—ITS SYMPTOMS AND TREATMENT.

BY ROGER G. PERKINS, M. D.

Senior Assistant Physician, New York Lunatic Asylum, Blackwell's Island.

(*Continued from page twenty-five.*)

The general physical health of patients suffering from partial mania, is good in many cases. Anæmia is the prevailing physical derangement, where any exists. The heat of head is sometimes noticeable on the advent of excitement, and the pulse is not unfrequently quickened.

Under this class it is proposed to speak of puerperal mania, though often the patient is *generally*, instead of partially insane. This form of mental derangement, so peculiarly interesting, inasmuch as it involves the happiness of so many and separate ties so closely woven, is connected invariably, as far as our observation gees, with a state of anæmia, and high, general, nervous excitement of the physical system. The onset of the disease may occur during gestation, parturition, or lactation. When seen in connection with gestation, it is easily confounded with an exaggerated hysteria; in fact, when occuruing at this or any other period, it may be considered as a disease arising from hyperæsthesia, and therefore belonging to the same class as simple hysteria.

Had tendency to insanity been thought of, perhaps the tonics indicated by the anæmia might have been suspended, lest they might add to the violence of the paroxysm. The cessation of tonics, in these cases, invariably proves disastrous. It is not proper that the consideration of the symptoms present in this form should be left without more marked reference to the propensity generally existing, to use filthy and blasphemous language—particularly the former. Ideas the most repulsive are constantly expressed; and a wanton exposure of the person, with sometimes a great desire for sexual intercourse, in many cases form the principal symptoms.—The continued congestion of the ganglia of procreation seems to react upon the cerebrum, and the language is influenced by it. It may be observed, in passing, that the same rule holds good in relation to the congestion of the ganglia of digestion; this state of things also reacting upon the cerebrum, ideas of foreign substances in the stomach are born, which ideas will continue until the cause is removed.

Typhomania is a form of insanity which has only of late attracted the attention of psychologists. As the name would seem to indicate, it consists of the worst symptoms of general mania, accompanied with a marked typhoid tendency. There is unwillingness to eat or drink; and all food as well as medicine must be forced upon the patient by means of "the wooden spoon," a description of which will be found in that part of the paper which is devoted to the consideration of treatment.

The physical strength, when put in opposition to the attendants, where medicine or food is to be given, is oftentimes very remarkable. In the cases I have seen, there has been no suicidal attempt. The general employment of the "*camisole*" may, however, account for this fact. The mortality is very great, though more success has followed the exhibition of stimulants than any other treatment. Depletion would undoubtedly destroy the patient.

In regard to the nature of this form of mania, some have considered it an aggravated febrile delirium, some as mania complicated with typhus fever, others again as an hitherto undescribed form of mania. Our experience of typhus and mania in their ordinary and extraordinary forms, permit us to hold only the opinion that it is truly a form of mania, bearing the same relation to that disease as the pneumonia typhoides does to the pure pneumonia.

II. Having thus cursorily glanced at the symptoms of one of the principal forms of insanity, and having followed any peculiarities into the class in which they exist, it is proposed to proceed to the next form—*melancholia.*

Of all the physical causes, productive of melancholy the most frequent is dyspepsia among men, and disease of the cervix-uteri among women.

Diseases of the liver, of themselves, as well as in their connections with general digestive derangement, are a frequent cause.—Cancer, or even chronic inflammation of the stomach, are often present as causes; and hernia in all its forms, may also be classed in the same category. Hypochondriasis is the first step toward mleancholic insanity, in many instances; and when, at last, insanity is reached, the hallucinations are sufficiently ridiculous.

The condition of the melancholic is, whether the disease is of mental or physical origin, one appealing to our kindest sympathies.

It is seldom accompanied with violence, unless in opposition to medication and curative agents. When, however, violence occurs, the type of the disease is that of general or partial mania. Constantly hopeless and despairing; often weeping; sometimes gazing, in an apathetic indifference, upon vacancy; answering, when spoken to, in the most plaintive tones, and referring, at all times, to the cause of their heart-breaking sadness. This class of patients occupy a place in the world without *filling* it, live neither in the present or future—contemplating only the past. Sometimes in melancholia from hypochondriasis, amid tears and moans, you are informed that a serpent is within the stomach, gnawing at the vitals; that crabs, shoemakers, poodle-dogs, fire, glass bottles, poisons, stones, laudanum, handkerchiefs, iron hoops, swords, in short, any thing you please, wasps, men and women, pepper, acids, etc., are in the belly; in another direction is one who assures you his legs are glass, and his head is a pumpkin. Here is seen the intimate connection between the ganglion of digestion and the cerebrum—the irritation of the one calling upon the other for sympathy.

III. *Dementia*, the third form into which we have divided insanity, consists either in complete incoherence or complete inaction (amentia.) The incoherence is too great to allow of the presence of "fixed ideas"—hallucinations—in the one case; and the total want of mental action, accounts for their absence in the other.—Dementia is rarely acute. It most frequently follows upon acute general mania, and treads fast after the melancholia of the affections. It is also a *sequela* of the melancholia incident upon physical disease, though not so commonly as of the other form, unless the brain is involved in the organic lesion.

A patient having dementia, not unfrequently has moments of acute mania; sometimes those maniacal periods extend over a longer space of time, and for a few days the exaltation may be a marked symptom. Dementia, however, soon follows, and the patient is as abject and stupid as ever: any hopes formed upon those sudden changes are seldom destined to be fulfilled.

This form of insanity has three stages: loss of memory, loss of comprehension and reason, and loss of instinct. The three stages merge into one another, and it often occurs that the transition from one to another is not noticed. In the first stage, the loss of memory not interfering with the reason, that faculty is only slightly affected; provided the premises are constantly before the patient, his reasoning is not deficient: thus, a game of chess, or draughts, as the patient can have the situation of the men before him continually, is played by a patient in the first stages of dementia with a semblance of the skill which belonged to him before his illness; but if two facts are presented for his comparison, he forgets one in the study of the other. This stage may continue for some time, and, perhaps, the patient may never pass through the other forms of the disease.

In this stage, dementia is considered curable, in some instances; it is always, at this time, treated with hope. The second stage gradually makes itself prominent, and the patient is seen no longer to take interest in things about him, however agreeable they may have been formerly: with his hands in his pockets, or with arms folded across his breast, he stands stock still, his chin resting upon the top of the sternum, and his eyes cast to the ground; all day, through all kinds of weather, he would, perhaps, remain in this position, unless moved by his attendants. The most amusing incidents, the most moving events, pictures, music, &c., fail to arouse him, except with great difficulty. If the head is raised, by force, from its forward inclination, by the hand of the physician, and patient is addressed in a loud voice, the expressionless features do not respond, though the lips may commence an inarticulate, meaningless, reply. The extremities become swollen and œdematous; the circulation is languid; bowels are more or less costive; while the appetite is often voracious; and a desperate inactivity settles upon the whole mental and physical man.

We pass rapidly from the painful consideration of this stage, only remarking, perhaps unnecessarily, that hardly any cures are ever known from its first onset. Death generally frees the unfortunate from his disease. The cases of this form, in many lunatic asylums, are numerous; as the accumulation of incurables generally die in this state.

IV. *Moral Insanity*, is a disease of the affections and feelings: in it the mind is not necessarily involved. When it occurs in connection with partial mania, the cases are most difficult and unmanageable. When occuring by itself, antipathies of an exaggerated character, and the most utter disregard of truth, are prominent symptoms. With the most unblushing effrontery, the patient will demand our belief of false assertions—perhaps likely to be true—to the injury of others; and, with the perfect knowledge on his part of the falsity of the accusations, tell a consistent story against those for whom he has an antipathy. When asked as to the reason of his antipathy, he does not know why he cherishes his hate, and can scarcely be got to acknowledge his lie. In the progress of the disease he becomes filty, insolent, and overbearing, and seems driven on by the Spirit of Evil to do evil simply for evil's sake. An individual laboring under this form of disease, may pluck a flower and trample it in the earth, for no other reason than because it is beautiful, and may afford enjoyment to others; he may injure the fair fame of a woman, because she is virtuous; or, he may hate the wife of his bosom, because of her fidelity. In the advanced stages of this disease, there is a disposition to rend, and the habits are of the most filthy and revolting character. In purely moral insanity, no delusions exist. Patients have been known to excuse themselves from their habits, on the ground of insanity.

The treatment of insanity is both medical and moral. It is of the medical we shall principally speak at this time. In the management of this disease, we are guided by the same general principles as in that of every other. Our first step is to remove the cause. If that cause is a physical disease, its treatment, and if possible, its cure, is our first duty. If physical symptoms are not at first prominent, they are to be sought for with great care: in almost every case they exist, and demand treatment. It should be borne in mind that insanity is but a symptom, and as such, its origin should be discovered at once.

That the desired treatment may be carefully carried out, the first object of the physician, on being called to a case of mania for its treatment, should be to procure a strong, quiet, trustworthy, and patient man, as attendant. This person, upon whom great responsibility rests, should have the virtue of consistent firmness with unremitting kindness. He must be entirely under the control of the physician, and must in the cases of mania never lose sight of the patient under any circumstances whatever. He must be provided with a camisole, a "wooden spoon," and additional aid, if he requires it, to enforce the physician's orders. This camisole (French for waistcoat) is a jacket made from sail-cloth. The sleeves are two feet longer than a man's arms. The jacket is fastened behind with strong buckles and straps. The arms, passed into the sleeves, are crossed upon the chest, and the two long sleeves are brought behind and tied together. When a patient is within this enclosure, his arms are confined, not at all uncomfortably; and, the only opening of the camisole being behind, by no possible manœuvering can the apparatus be removed by the patient. In this condition, the idea of his helplessness will soon take possession of the patient's mind, and he will often submit unwillingly to take the medicine ordered, as well as the food before refused. If, however, he persists in his refusal of food and medicine, he must be placed gently upon a mattress, his face uppermost, of course, and the wooden spoon used to administer what has been ordered. The spoon is a very simple, but very effectual contrivance. It consists of a spoon made of wood, in all respects resembling the ordinary form; the handle of it is hollow, and the cavity within extends to the point of the bowl. The spoon, when used, is to be forced between the teeth, and its bowl to be carried back until its point has reached near the root of the tongue. The fluid to be administered is then poured through the hollow handle, from the mouth of a coffee-pot or pitcher; and the patient is compelled to swallow, as the fluid reaches the portion of the pharynx controlled by the reflex system of nerves.

Having put himself in the possession of those means for the thorough accomplishment of his plan of treatment, it becomes the physician's duty to form his plans, secure in the conviction of his ability to execute. In speaking of the *therapeutics*, the question of blood-letting at once presents itself. *There is hardly any form of insanity in which bleeding is admissable.* In every case in which it has been employed within the writer's knowledge, it has proved highly injurious; and, in one case, perhaps utterly destroyed the hope of cure, which might otherwise have been cherished. If a strong, plethoric, and physically healthy man becomes the subject of acute mania, if the head is hot, eyes injected, and face flushed, pulse full and strong, and at the same time if the patient is violent and noisy, the physician would naturally be tempted to a trial of general blood-letting. Let him on no account yield to his inclination. The voice of the specialists in this department is raised strongly in opposition to such a course, and nothing but evil has resulted from this manner of treatment in the cases observed.

The patient will require all his natural strength during his convalescence to avoid falling into dementia. Again, other and powerful agents are at hand to lessen the violence of the sick man, which are equally immediate in their effects, and, at the same time, conductive to a permanet cure. Of these we at once proceed to speak.

Hot and prolonged baths, with ice-water to the head, are the most serviceable means which can be used in the *suppression of the violence* of acute mania, and, at the same time, are excellent means of cure. Let the patient be placed in a bath raised to 98 deg. or over, and while in the bath-tub, let cloths dripping with ice-water be kept constantly applied to the head, the hair on which has been cut very close to the scalp. In a few moments, perhaps it may be half an hour, the patient begins to grow calm, and perhaps will express himself pleased with his treatment, and desire its continuance. The warmth of the bath is to be kept up by fresh additions of hot water, as they may be required; and the cold applications to the head must be continued without intermission.—The length of time this bath is to be continued, will vary with the patient's pulse and manner. In many cases the pulse will change to nearly or quite the normal standard, before the bath should be suspended. In Germany, maniacs are not unfrequently kept in this warm bath, with cold applications to the head, for eighteen consecutive hours on following days. It is said that many patients are cured in three weeks by this means. In the treatment of acute mania, and in chronic mania also, when the physical system has but slightly suffered, we have witnessed the most surprising effects from the use of this agent. Patients which, without its exhibition, would have been violent, and necessarily confined in cells for weeks and months, have been so benefited by the bath as prescribed above, as to be reasonable and responsible, trusted and trusting, after but a few applications.

Counter-irritation to the nape of the neck, from the employment of the cantharidal collodion (a most excellent invention for insane hospitals), the tartar-emetic ointment, Granville's lotion, &c., &c., are often of great service. Setons are too slow in their effects to be of much use in acute mania. In the other forms of insanity they are sometimes highly beneficial. In the application to the scalp of irritating ointment, erysipelas is to be feared; and this plan is not often followed with us. In cases of partial mania, however, the tartar-emetic ointment has been used over the site of the organs of Combativeness and Destruction, where these organs seemed concerned in the disease (from the form of the hallucinations and the course of the habit); and benefit has resulted in some instances. Tartar emetic administered internally, as a means for obtaining quiet, is often successful. The mercurial purge of calomel and jalap, with one or two grains of tart. ant., is generally administered to patients when the raving mania is accompanied by a disordered stomach. It is often well to open the course of treatment with this evacuant.

In many cases, the use of such remedies as are recommended above, is by no means admissible. Anæmia is a common accompaniment to mania, and a state of the system decidedly below par is too often met with. In these cases the only, and in a great proportion of them the *efficient*, remedies are tonics and sedatives —conium and iron, in large and continued doses (after the system has been, by proper alterative medicines, prepared to respond to their action)—are used with great success. Morphine, or any preparation of opium is much used, and with marked good results. Now and then, a blister may be applied to the nape of the neck, but only for a short time; it is soon healed up. The best food is to be allowed the patient; and healthful exercise in the open air, or in an enclosed verandah, always with his attendant, is advisable.

Puerperal mania, as we have seen it, has demanded the full exhibition of tonics and sedatives. The conium and iron mentioned above are those generally employed. The same principles which govern the treatment of general are held in the management of partial mania. If the habits of the patient seem to require it, he must be treated with depressants; if the contrary, tonics and opium are specially indicated.

Opium, in partial mania, seems sometimes to act upon the brain

*alteratively.* The patient is given three half-grains of morphine per diem, for weeks and months together. It is sometimes gradually increased, and after a while the hallucinations are seen to be disappearing. They finally leave the patient altogether, and the medication is gradually suspended. In partial mania, great aid is received from moral treatment, of which more hereafter.

Typhomania is to be treated in precisely the same manner as typhus fever, in many respects. Morphine is, however, to be used in this form of disease, to lessen the extreme violence. Brandy and milk, beef tea, &c., &c., are always to be exhibited ; and the full use of tonics must be employed during the convalescence.

In *melancholia*, the treatment must be different in the two forms. When the disease is the result of exaggerated affection, the influences brought to bear upon it are chiefly moral.

The exhibition of morphine as an alterative in this form, however, is often of the greatest benefit. Its effects upon the physical system in producing quiet, and in removing that state generally known by the term nervousness, are very evident. Its influence upon the mind in producing oftentimes an entire change in the current of ideas, is well known. In place of sombre and mournful thoughts, the mind of the patient, through the faculty of imagination, is led to contemplate beautiful visions, and gradually induced to prefer them as subjects of thought. His grief is forgotten amid the realms of ideal beauty through which he wanders ; and, no longer constant to the object of his sadness, the patient becomes more cheerful, and, many times, gradually recovers.

In *dementia* the course of treatment also includes many appeals to the "*morale*" of the patient. The chief medication is found in the means best calculated to establish the general health. As may be inferred, the marked intonicity of the reflex system, almost always present, demands attention. The different preparations of nux vomica, in small but long-continued doses, the canabis indica in a few instances, iron, cinchona, the vegetable tonics and stimulants, are not to be omitted.

Setons, in the nape of the neck, which shall be kept open for weeks and months, are, in this form of insanity, of more use than any other. They act in two ways. They form an object upon which the attention of the patient is often fixed (which is not to be slightly estimated in dementia), and, at the same time, they act as a continued slight counter-irritation to the encephalon. The drain from the system established by them, if supplied with the materials for good blood, is of marked service, from its indirect alterative effect.

Morphine is rarely used in dementia, the lack of its indication is at once perceived. The system is already sufficiently sluggish.—Mercurials are sometimes indicated, as well as iodine also.

*Moral insanity* may, perhaps, be sometimes treated with antimony, if the system is in such a state as to admit of its use. If employed, it should be continued for a moderate length of time, to allow a complete manifestation of its effects. Morphine, as an antidote to the physical agitation, has been, in some cases, followed by improvement.

By furnishing, through its effects on the cerebrum, new food for thought, it may remove the morbid tendency to waste the energies upon falsehood and wickedness. In this form of derangement, as has been said above, there are no *bona fide* hallucinations.

The moral faculties are alone involved, and counter-irritation, employed with this view, may, perhaps, influence the disease.—Constant occupation, travelling, manual labor, and radical change in the daily associations of the patient, must result in benefit. Toward one affected with *moral mania*, the bearing of the physician should be that of a calm, high, censor of his actions.

Before leaving the subject of medical treatment, distinct reference should be made to the use of *etherization*. Dr. Ray, of the Butler Hospital, at Providence, has lately read a paper upon this subject, before the convention of Medical Superintendents of Lunatic Asylums, in which he highly commends its employment. We are beginning to exhibit it, and hope for good results. After the patient is, in some degree, under the influence of opium, ether will often change the drowsy stupor attending the full dose of this drug into sound and apparently healthy sleep. A good night's rest, obtained night after night, by these, or any other means, cannot but benefit the patient extremely.

*Suicidal cases*, always the objects of unceasing care, but, particularly to be watched during the hours of darkness, are to receive great benefit from this new form of treatment. They are quietly sleeping, with, perhaps, happy dreams (when under the combined influence of opium and ether), during the period in which the attempt at self-destruction is most to be feared. Night after night of unbroken sleep changes the current of their thoughts, and, with moral treatment suited to their peculiar state, their depression is oftentimes entirely removed.

Moral treatment is best conducted, in almost all instances, by persons who have not known the patient before his illness. It is on this account, as well as for the purpose of removing the patient from scenes constantly reminding him of his ordinary life and business, that the insane are generally more successfully treated in asylums. The psychologist, in his bearing toward those under his charge, may, and should, exhibit the feeling of sympathy which he has at heart for their misfortunes. He is always the same affectionate, firm, *truthful*, sympathizing friend. With quiet patience he listens to all their complaints, and by his kind manner and expressed interest (to some degree) assuages their sorrows. A subdued earnestness, with evident intensity of thought, and a certain deliberateness of movement and expression, seem to gain the confidence of the wandering mind in most cases.

It is a matter in some dispute if the patient is to be informed that he is considered insane. Among the Germans, it is not uncommon to do this, and the practice at Utica is also of this character; our own impressions are greatly in favor of this plan, and we have known several instances in which a patient (under medical treatment also) has been *reasoned out* of his delusions. It is many times better *not to reason* or argue with the insane, who may think you are the insane man, rather than he; and, also, because a man who disputes with another places himself upon a level with his opponent.

If one simply states that the subject of the delusion is one on which he cannot agree with his patient; that he most fully *denies its truth*, but is *not disposed to talk* upon the matter, he will, in almost all cases, retain the patient's confidence; and, as he is considered somewhat as a superior being, his judgment is not unusually conceded to. It is hardly ever improper to inform a patient that the actions of his mind are deranged, but there are some instances when it is not necessary to refer to it. Employment is the greatest moral means possessed by the physician in the treatment of the insane. It is better that the nature of this employment should be of the character of the previous habits of the patient; but out-door occupation is by far the most serviceable. As an instance of what employment may do for even the most excited patients, I may be allowed to state that on a recent visit to the New York State Asylum, the most excited and noisy patients were found sitting in a circle round a basket, shelling green peas. They were quite busy, and hardly looked up until the presence of a stranger attracted their attention. The restoration of several lunatics to their senses by a Scotch farmer, who employed them upon his farm because they cost less than men who were not insane, was, perhaps, the first thing which attracted general attention to the use of farm-labor in the treatment of this disease. It is every day affording proofs of its great benefit, in almost every asylum in the country.

Employment at the trade of the patient, if the employment is a healthy one, is an excellent means of cure, when this moral is united with proper medical treatment. There are certain patients whose incoherence and violence are so great as seemingly to preclude the idea of any steady occupation; but unremitting perseverance on the part of the attendant will surely bring its reward, and the patient and the physician will each wonder at the amount accomplished.

Amusements should hardly ever be allowed to fill the whole time of the insane. They are generally to be employed as offsets to the wholesome fatigue engendered by some useful occupation. In speaking of the *moral treatment* of special cases of lunacy (the unexpected length of this paper warns us to touch lightly upon the subject) it may be remarked, that the manner of addressing the patient must differ with the character of the person and with his special delusion. The innumerable little incidents resorted to for the purpose of drawing the attention of the demented ; the many little means used to excite the cheerfulness of the melancholic ; and the many soothing influences brought to bear upon the excitement of the maniac, to produce quiet and calmnesss, are not to be mentioned in this place. They will suggest themselves in the treatment of the patient, from the circumstances with which he may be surrounded.

Before closing this paper, my duty would be hardly half done, did I not refer to the advantages of sending the insane to an asylum as soon as possible. The symptoms and plans of treatment which have been laid down, are for the purpose of aiding those who require such assistance in the diagnosis of the different forms, and in the treatment of the disease in those cases when circumstances will not permit of sending the patient to a public institution, and in those cases in which unavoidable delay in conveying the patient to an asylum is met with.—*American Medical Monthly.*

